# lncRNAs as prognostic molecular biomarkers in hepatocellular carcinoma: a systematic review and meta-analysis

**DOI:** 10.18632/oncotarget.19559

**Published:** 2017-07-25

**Authors:** Chuqian Zheng, Xiuxia Liu, Leifeng Chen, Zheng Xu, Jianghua Shao

**Affiliations:** ^1^ Department of General Surgery, Second Affiliated Hospital of Nanchang University, Nanchang, 330000, China; ^2^ Jiangxi Province Key Laboratory of Molecular Medicine, Nanchang, 330000, China; ^3^ Jiangxi Province Engineering Research Center of Hepatobiliary Disease, Nanchang, 330000, China

**Keywords:** lncRNA, hepatocellular carcinoma (HCC), survival, prognostic, meta-analysis

## Abstract

The latest studies have shown that long non-coding RNAs (lncRNAs) may be considered markers as their expression levels were abnormal in cancer and can be used as a molecular biomarker for the potential assessment of cancer prognosis. In this study, we aimed to assess the prognostic value of lncRNA as marker of patients with hepatocellular carcinoma. We performed a detailed search of the PubMed and Embase databases for articles on the prognostic value of various lncRNAs in HCC. We then carefully extracted the relevant data from the articles, and we used the meta-analysis method to analyze these results; heterogeneity and publication bias were also evaluated. With 40 associative studies included, we found that high expression of 27 types of lncRNA was associated with a poor prognosis in HCC patients, and low expression of 18 types of lncRNAs was associated with a worse prognosis. Patients with higher lncRNA expression had significantly poor overall survival (OS; pooled HR, 1.25; 95% confidence interval [CI], 1.03–1.52) as well as significantly poor recurrence-free survival (RFS; pooled HR, 1.66; 95% CI, 1.26–2.17). Overexpression of lncRNAs may not meaningfully predict disease-free survival (DFS; pooled HR, 1.04; 95% CI, 0.52–2.07; *p* = 0.91). Our meta-analysis demonstrated that lncRNAs may serve as predictive biomarkers for cancer prognosis.

## INTRODUCTION

Liver cancer is the fifth most prevalent type of cancer affecting the global population, with mortality rate ranking third in the world [1, 2]. Its incidence remains highest in the developing world and is steadily increasing across the developed world [3]. Currently, patients diagnosed with early stage liver cancer usually choose surgical resection based on the location and size of tumor, while transcatheter arterial infusion (TAI) and transcatheter arterial embolization (TAE) are often adopted for intermediate or advanced stages of liver cancer that are difficult to be surgically removed [4]. Although surgical resection and radiochemotherapy have been found to be effective to some degree, radical cure of the liver cancer remains difficult, and the prognosis is also unsatisfying [5]. The expression of lncRNAs was always abnormal in liver cancer, and this can closely associated with the prognosis of cancer [6, 7]. The researchers found that the JPX and XIST levels were abnormal in HCC and associated with histological grade and tumor-node-metastasis stage. Therefore, it is of great clinical value to search for markers to help identify the cancer in the early stages, predict the prognosis, and develop new treatment strategies.

The latest studies have indicated that, as hepatoma-specific tumor markers, lncRNAs contribute to the diagnosis and treatment of liver cancer [8]. lncRNA is a class of non-coding RNAs with more than 200 biological functions. lncRNA expression has been found to be much higher in diffuse cancer cells than that in early cancer cells, and the specific expression of lncRNAs in tumors may be used as an effective biomarker for cancer diagnosis and postoperative observation. Many studies have identified the abnormal expression of many lncRNAs in hepatomas, such as highly upregulated in liver cancer (HULC), HOX transcript antisense *RNA* (HOTAIR), WD repeat containing antisense to TP53 (WRAP53), differentiation antagonizing non-protein coding RNA (DANCR), maternally expressed gene 3 (MEG3), ZEB1 antisense RNA 1 (ZEB1-AS1), ICAM-1-related (ICR), and small nucleolar RNA host gene 1 (SNHG1) [9–16]. The upregulation of HULC was first characterized in hepatocellular carcinoma. HULC regulates oncogenic mRNAs by promoting phosphorylation of YB-1, and then activating downstream signaling pathways, and finally accelerates the translation of these mRNAs to promote the process of tumorigenesis. Some lncRNAs are highly expressed in tumors, while others have low expression, and the relationship between lncRNAs and tumor prognosis remains controversial.

Overall, the aim of our study is to evaluate the value of all types of lncRNAs in the prognosis of hepatocellular carcinoma (HCC) patients.

## RESULTS

### Features of included literatures

**Figure 1:**
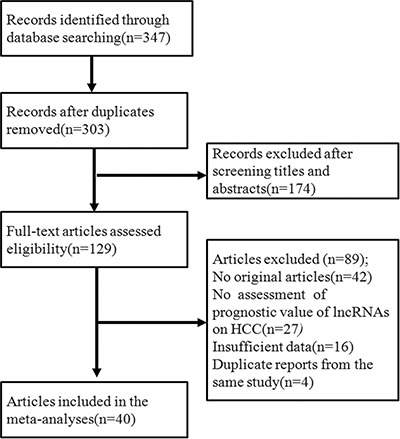
Flow diagram of the study selection process A final total of 40 articles were included for meta-analysis.

**Figure 2:**
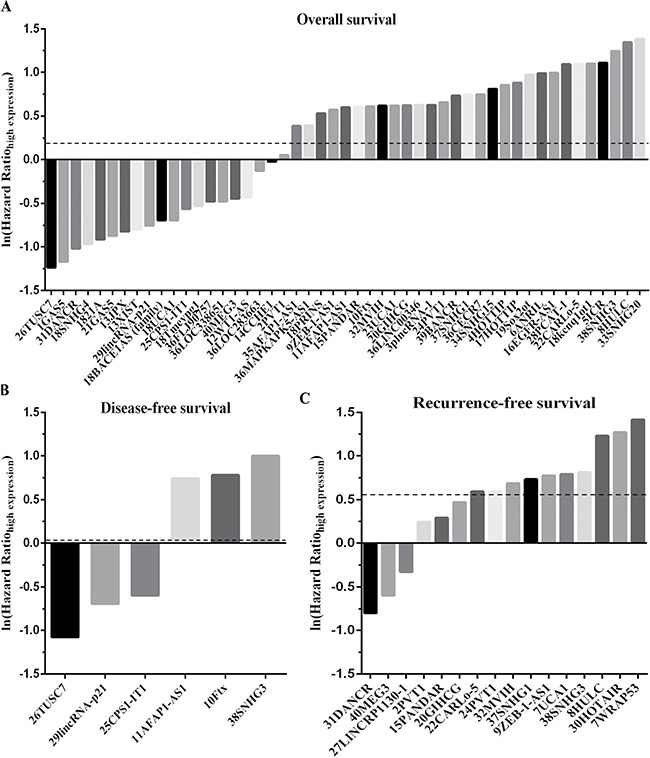
The histogram for hazard ratio (HR) and 95% confidence interval (95%CI) values of each lncRNA affected the survival rate of HCC (**A**) Overall survival (OS). (**B**) Disease-free survival (DFS). (**C**) Recurrence-free survival (RFS). (Take the 26TUSC7 for example, it contains two parts. The number of 26 represents the serial number among the 40 stuies, and the TUSC7 stand for the lncRNAs’ name).

Through a search of the PubMed and EMBASE databases, a total of 347 primary studies were obtained. After eliminating 307 papers that did not meet the selection criteria in the next screening, a final total of 40 articles were included in the study. The original data were extracted from these 40 papers for meta-analysis [9, 11, 12, 15, 25–40] and other airticles [10, 13, 14, 16, 41–56]. The flow chart of the literature screening is shown in Figure 1. Forty included articles were published between 2011 and 2016, and all of them were retrospective studies reporting the expression of 71 lncRNAs in HCC patients. The average sample size in these papers was 120. In most studies, lncRNAs were detected in the patient's plasma. All lncRNAs were measured using quantitative real-time polymerase chain reaction (PCR), with glyceraldehyde 3-phosphate dehydrogenase (GAPDH) or β-actin as the reference gene. Five independent reviewers assessed the 40 included articles according to the method mentioned above, with the quality remaining moderate. Supplementary Table 1 provides more information on the assessment. Twenty-nine of the included articles directly reported the relationship between high-level lncRNAs and the survival rate, while 11 studies reported the relationship between low-level lncRNAs and the survival rate. Their HR value and 95% CIs are shown in Supplementary Table 2 and Figure 2 shows that an HR > 1 implied worse survival for the group with elevated lncRNA expression. Conversely, an HR < 1 represented worse survival for the group with decreased lncRNA expression.

### lncRNAs and prognosis

**Figure 3:**
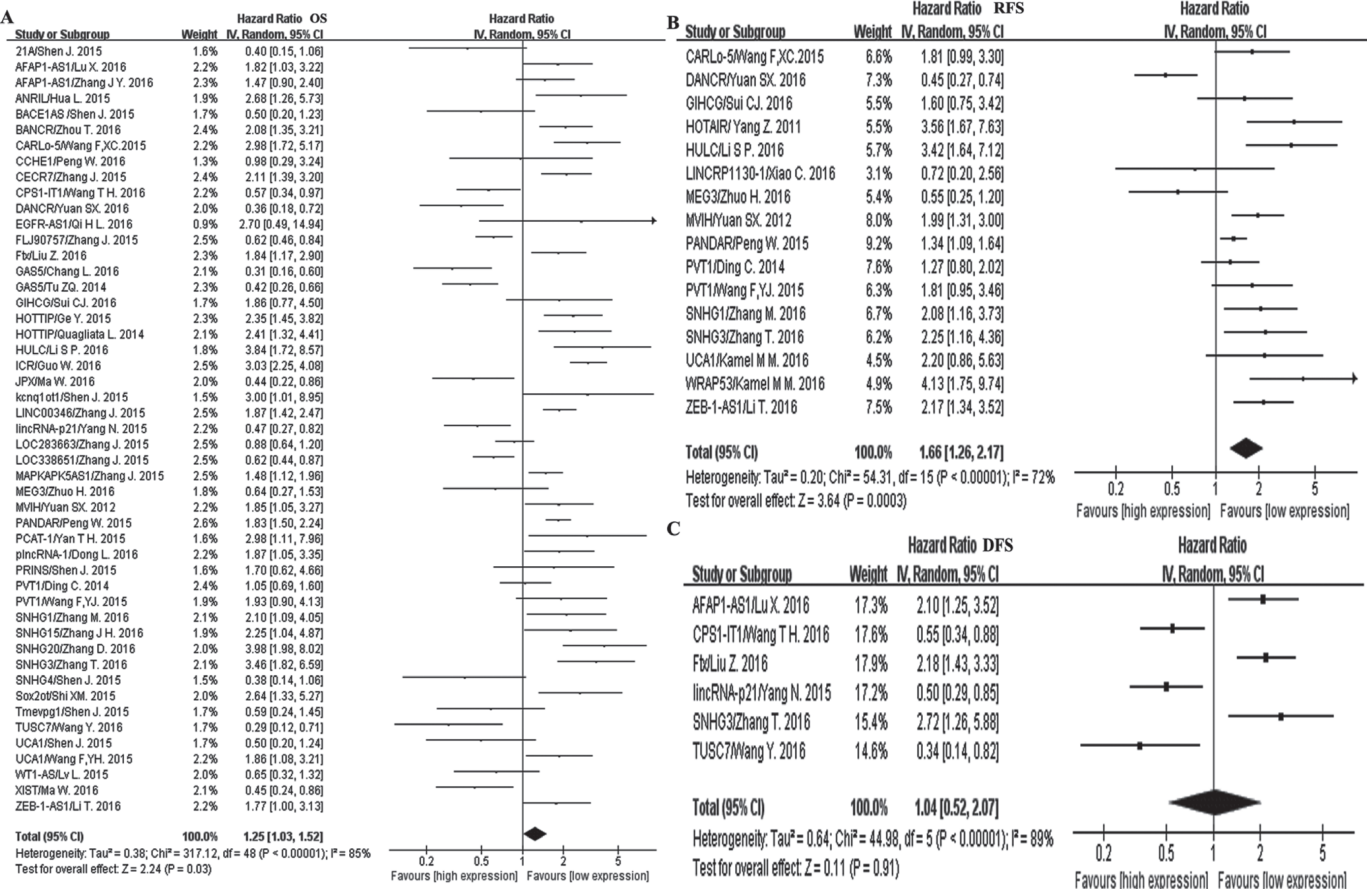
Forest plots of the studies to assess the pooled hazard ratio (HR) and 95% confidence interval (95%CI) values of lncRNAs (**A**) Overall survival (OS). (**B**) Disease-free survival (DFS). (**C**) Recurrence-free survival (RFS).

Forty articles addressed the relationship between lncRNA expression and HCC prognosis. Taking into account the significant heterogeneity, we chose the random-effects model for our meta-analysis. We summarized the high expression and low expression of lncRNAs in the Supplementary Table 3. The pooled HR was 1.25 (95% CI, 1.03–1.52) for 19 of the lncRNAs with lower expression level and 30 of the lncRNAs with higher expression level, indicating that higher lncRNA expression levels predicate poorer OS for HCC patients (*p* = 0.03) (Figure 3A). Fifteen studies comprising 15 kinds of lncRNAs evaluated HCC RFS, and we found a significant association between the lncRNA expression level and RFS (pooled HR, 1.66; 95% CI, 1.26–2.17; Figure 3B), which showed that higher lncRNA expression represents a worse prognosis. Additionally, 6 studies evaluated DFS for 6 types of lncRNAs, and its pooled HR was 1.04 (95% CI, 0.52–2.17), but the results were not significant (*p* = 0.91, Figure 3C). Moreover, in studying the association between OS and lncRNAs, the noteworthy consideration of the 2 special gene families is due to that there is a debate on whether SNHG and UCA1 are oncogenes or tumor suppressor genes. After individually studying these genes, we found that the high expression of SNHG had worse prognosis for OS, while the use of UCA1 for prognosis of OS was meaningless. These findings are illustrated in Supplementary Figure 1.

**Figure 4:**
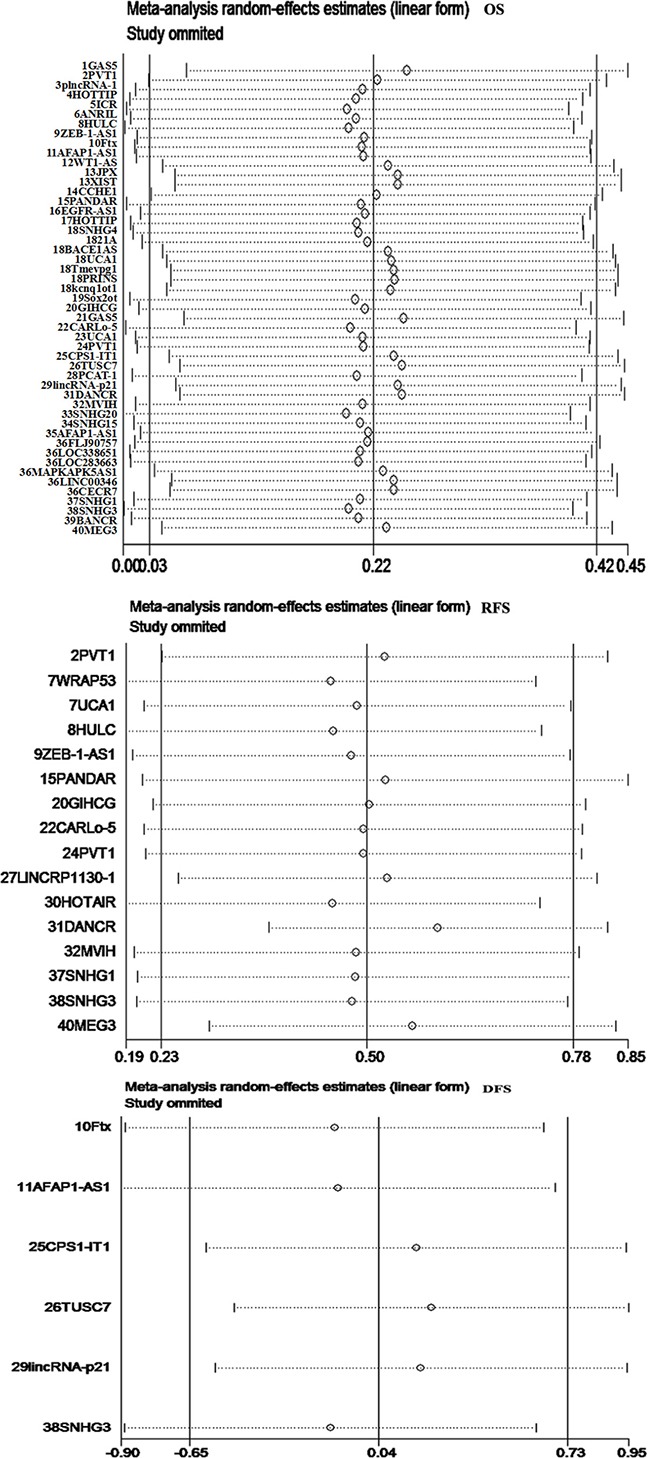
Sensitivity analysis to evaluate the stability and reliability of meta-analysis results There was no change in the pooled HR results after excluding any research data of one study.

There was significant heterogeneity in the meta-analysis for OS, RFS, and DFS (I^2^ > 50%, *p* < 0.01). To evaluate the stability and reliability of the meta-analysis results, we performed sensitivity analysis, which indicated that there was no change in the pooled HRs results after excluding research data of one study (Figure 4).

### Publication bias

**Figure 5:**
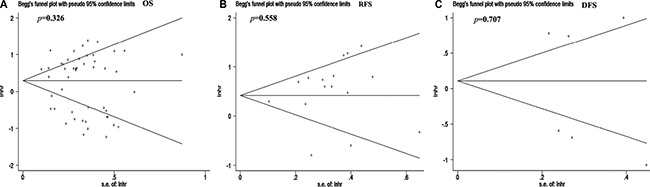
Use of Begg's funnel plot to evaluate publication bias of the included studies There was no significant publication bias for all the included studies. (**A**) Overall survival (OS). (**B**) Disease-free survival (DFS). (**C**) Recurrence-free survival (RFS).

We used Begg's test and Egger's test to evaluate the publication bias. As shown in Figure 5, the Begg's funnel plot tests for OS, RFS, and DFS had *p* values of 0.326, 0.558, and 0.707, respectively, and Egger's tests for OS, RFS, and DFS had *p* values of 0.325, 0.365, and 0.691, shown in Supplementary Figure 2. Therefore, we can conclude that there is no significant publication bias among the included studies.

## DISCUSSION

Although the development of diagnostic methods and surgical techniques in recent years has remarkably improved the prognosis of HCC patients, the 5-year survival rate for advanced HCC remains poor [57]. However, several noninvasive blood markers are now available for HCC detection [58]. For example, AFP, gamma-glutamyl transferase (GGT), des-γ-carboxyprothrombin (DCP), human growth factor (HGF), HSP70, and interleukin 6 (IL-6) are currently used in detection of HCC. Additionally, many scholars are interested in developing a holistic comprehensive study of the relationship between one biomarker of tumor diagnosis and prognosis by meta-analysis [59].

Many lncRNAs have been found to change in HCC, and the expression level of lncRNAs is related to tumor size, TNM stage, and prognosis [60]. LncRNAs interact with miRs to control the invasiveness and metastasis of HCC cells [61]. As a key factor in the development and progression of HCC, overexpression of carcinogenic lncRNAs and suppression of tumor suppressor lncRNAs control the invasion, metastasis, cycle, and angiogenesis of liver cancer cells [62]. To validate the accuracy of the reported lncRNAs as prognostic molecular markers for HCC, we systematically reviewed and meta-analyzed the currently available papers on lncRNAs and evaluated the value of lncRNAs as prognostic markers. Our study is the first extensive report focusing on this association, in which 40 studies were analyzed, and 71 types of lncRNAs involved in the survival analysis of HCC were compared.

In this meta-analysis, we found that inconsistent expression level of overall lncRNAs in the blood has a positive statistical significance. The results of the meta-analysis indicated that lncRNA expression levels are a promising biomarker to predict survival in patients with HCC. Compared with patients with low expression levels of lncRNAs, patients with increased expression levels of lncRNAs had a 1.25-fold higher risk of poor OS, a 1.66-fold higher risk of RFS, and an insignificant risk of DFS. Heterogeneity is an important reference factor for meta-analysis. Forest plots and I^2^ showed the existence of heterogeneity. Hence, sensitivity analysis and meta-regression analysis were used to determine the possible causes of heterogeneity. Meta-regression analysis revealed *p* values greater than 0.05 for all specified covariates, indicating that the heterogeneity of the sources of uncertainty had been identified. Additionally, different cutoff values among the included studies may also be a potential source of heterogeneity. More well-designed studies with large sample sizes are needed to clarify this issue and further explore the relevant mechanisms.

Like others studies, we used the same indicators(OS DFS RFS) to evaluate the prognostic, the same method to assess the risk of bias, the same test model, the same system analysis software. Other scholars were focused on one or several lncRNAs in their studies, However, we analyzed 71 types of lncRNAs in our study. Interestingly, we found that SNHG and UCA1 which two kinds of lncRNAs results are more special. In the tumor, these two lncRNAs are still controversial to be known as Oncogene or tumor suppressor gene. Our results show that the high expression of SNHG had worse prognosis for OS, while the use of UCA1 for prognosis of OS was meaningless.

This systematic review had several important strengths. First, we conducted a relatively thorough systematic search and applied a comprehensive analytic approach to evaluate the prognostic value of lncRNAs in patients with HCC. Second, the study's methods were rigorous and followed the guidelines for conducting and reporting systematic reviews. However, there were also some limitations in our analysis, such as the considerable heterogeneity. Meta-regression and sensitivity analyses were applied, but the results could not fully explain the observed heterogeneity. LncRNAs can be evaluated by noninvasive method to evaluation tumor invasion and migration ability, combined with clinical tumor size, TNM stage, which provides the prognostic biomarker in HCC.

## MATERIALS AND METHODS

### Systematic review

This meta-analysis was carried out in accordance with the Preferred Reporting Items for Systematic Reviews and Meta-Analyses (PRISMA) statement [17].

### Eligibility criteria

The main inclusion criteria for the studies involved the prognosis of HCC, measuring the expression of specific lncRNAs in tissue or serum, and studying their association with survival outcome. Survival outcome was further explored by considering hazard ratio (HR) with confidence interval (CI), HR with *P* value, and Kaplan–Meier curves or was obtained by contacting the corresponding author [18]. Articles were excluded for the following reasons: (1) not written in English; (2) case reports, letters, or review articles; (3) sample size of less than 20 cases; (4) concerned genetic alterations of lncRNAs, including polymorphisms or methylation patterns; and (5) lack of sufficient data for estimating HRs and their 95% CIs. When duplicate studies were retrieved, we included the most informative and recent article. The articles that fulfilled all selection criteria were then processed for data extraction. Five individual researchers independently assessed the eligibility of the retrieved articles.

### Information sources

We conducted a meta-analysis of all studies of lncRNAs in HCC up to Nov 20, 2016. A literature search was performed on the PubMed and Embase databases for studies that analyzed associations between lncRNAs and prognosis in HCC patients before November 2016. The following search terms were used: (lncRNA OR Long non-coding RNA) AND (cancer OR tumor OR neoplasm OR malignant OR metastasis OR carcinoma OR hepatoma cell carcinoma OR HCC) AND (liver or hepatocyte) AND (prognosis OR prognostic OR survival OR outcome OR mortality). Electronic searches were supplemented by scanning reference lists of articles identified for all relevant studies (including review articles), by manual searching of relevant journals, and by correspondence with study investigators. Each study was assessed for inclusion by five reviewers independently, and discrepancies within the reviewing pair were resolved by discussion.

### Study selection

We focused on the titles and the summaries of the articles in detail. After the initial screening, we read the full text again and compared the article to our inclusion criteria. All data were extracted by the five authors, and all the retrieved information of the included studies was integrated into the final form. According to PRISMA guidelines, we have prepared the following screening information: (1) publication information: lncRNA name, author name, publication year; (2) the study type is randomized controlled study; (3) measurement of lncRNA expression; and (4) HRs of elevated microRNAs (miRs) for OS, DFS, and RFS as well as their 95% CIs and *P* values.

### Risk of bias in studies

For prognostic studies, the Newcastle-Ottawa scale (NOS) was applied to assess the risk of bias and the criteria for reporting observational studies to complete the methodologic evaluation [19]. These scales were used to allocate a maximum of nine stars for quality of selection, comparability, exposure, and outcome of study participants. Studies with six or more stars were rated as high-quality.

### Statistical analysis

All statistical analyses were performed by Review Manager 5.2, STATA 12.0, and SPSS 22.0 statistical software. Cochran's Q test and Higgins I-squared statistic were used to test the heterogeneity among the combined HRs [20]. The results come from the use of fixed-effect models or random-effect models. Fixed-effect models (*P* > 0.1 and I^2^ < 50%) demonstrate that the differences between the results of various studies are due to chance. Random-effect models (*P* < 0.1 or I^2^ > 50%) demonstrate that there is significant heterogeneity between the studies. When heterogeneity is absent, a fixed-effect model is considered more preferable than a random-effect model [21]. However, if heterogeneity is present, the use of a random-effect model is better [22].

Prognostic evaluation was assessed by HR, which was used to measure the prognostic performance of lncRNAs in our included studies.

### Publication bias

Begg funnel plots and Egger linear regression tests were used to assess possible publication bias [23, 24]. All analyses were performed using STATA version 12.0. A *p* < 0.05 was considered statistically significant.

## CONCLUSIONS

Despite the limitations described above, our comprehensive systematic review and meta-analysis revealed that lncRNAs could be promising, convenient, and potentially non-invasive prognostic markers in HCC. However, to draw a convincing conclusion on the value of these novel biomarkers for the prognosis of HCC, an appropriate and unified method should be established and applied.

This work was supported by grants from the National Natural Science Foundation of China (81560475 and 81460453), the Project of Jiangxi Provincial Department of Science and Technology (20152ACB20020 and 20171BAB215024) and the advantage innovation team of Jiangxi Province(20153BCB24004)

## SUPPLEMENTARY MATERIALS FIGURES AND TABLES






