# Supplementary material for: lncRNAs as prognostic molecular biomarkers in hepatocellular carcinoma: a systematic review and meta-analysis
Source: Oncotarget. 2017 Jul 25;8(35):59638–47. doi: 10.18632/oncotarget.19559 (PMC5601763; doi:10.18632/oncotarget.19559)
Supplement: Supplementary file 1 [file oncotarget-08-59638-s001.pdf]

lncRNAs as prognostic molecular biomarkers in hepatocellular carcinoma: a systematic review and meta-analysis

SUPPLEMENTARY MATERIALS

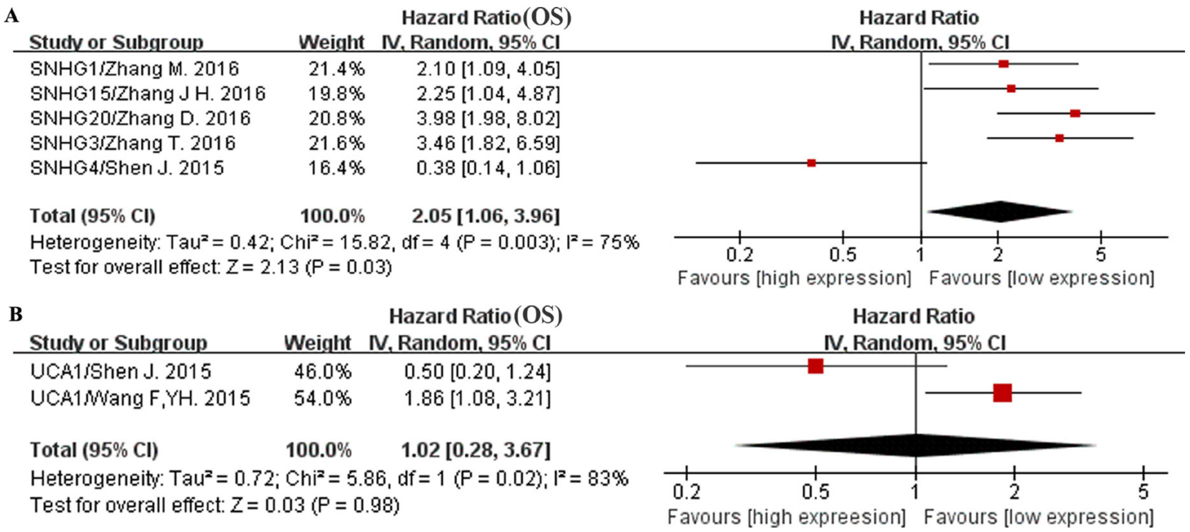

Supplementary Figure 1: Forest plots of studies evaluating hazard ratios(HR) and 95% confidence interval (95%CI) values of SNHG and UCA1 expression. (**A**) SNHG, OS ($I^2 = 75\%$, $p = 0.003$). (**B**) UCA1,OS($I^2 > 83\%$, $p = 0.02$).

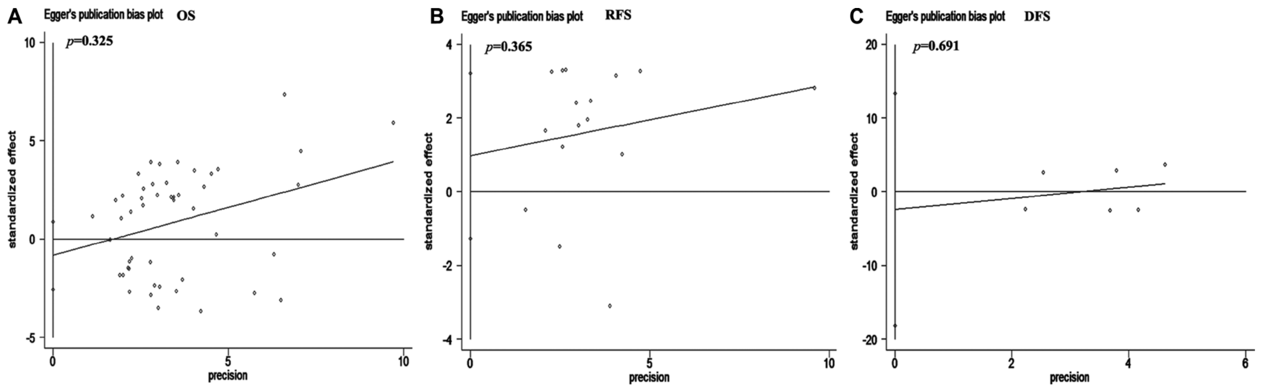

Supplementary Figure 2: Using Egger's funnel plot to evaluate these studies' publication bias. (**A**) Overall survival (OS). (**B**) Disease-free survival (DFS). (**C**) Recurrence-free survival (RFS).

Supplementary Table 1: Summary table of main characteristics for the eligible studies. See Supplementary_Table_1

Supplementary Table 2: Summary table of HRs and their 95%CI. See Supplementary_Table_2

Supplementary Table 3: The expression of lncRNAs

**21A/Shen J.2015**	**low expression**	**PVT1/Wang F,YJ.2015**	**high expression**
AFAP-AS1/Lu X. 2016	high expression	SNHG1/Zhang M.2016	high expression
AFAP-AS1/Zhang J Y. 2016	high expression	SNHG15/Zhang JH.2016	high expression
ANRIL/Hua L. 2015	high expression	SNHG20/Zhang D. 2016	high expression
BACE1AS/Shen J. 2015	low expression	SNHG3/Zhang T. 2016	high expression
BANCR/Zhou T. 2016	high expression	SNHG4/Shen J. 2015	low expression
CARLo-5/Wang F,XC. 2015	high expression	Sox2ot/Shi XM	high expression
CCHE1/Peng W. 2016	low expression	Tmevpg1/Shen J.2015	low expression
CECR7/Zhang J. 2015	high expression	TUSC7/Wang Y. 2016	low expression
CPS1-IT1/Wang TH. 2016	low expression	UCA1/Shen J. 2015	low expression
DANCR/Yuan SX. 2016	low expression	UCA1/Wang F,YH.2015	low expression
EGFR-AS1/Qi HL. 2016	high expression	WT1-AS/Lv L. 2015	low expression
FLJ90757/Zhang J. 2015	low expression	XIST/Ma W. 2016	low expression
Ftx/Liu Z. 2016	high expression	ZEB-1-AS1/Li T. 2016	high expression
GAS5/Chang L. 2016	low expression	CARLo-5/Wang F,XC. 2015	high expression
GAS5/Tu ZQ. 2014	low expression	DANCR/Yuan SX. 2016	low expression
GIHCG/Sui CJ. 2016	high expression	GIHCG/Sui CJ. 2016	high expression
HOTTIP/Ge Y. 2015	high expression	HOTAIR/Yang Z.2011	high expression
HOTTIP/Quagliata L.2014	high expression	HULC/Li SP. 2016	high expression
HULC/Li SP. 2016	high expression	LINCRP1130-1/Xiao C. 2016	low expression
ICR/Guo WX. 2016	high expression	MEG3/Zhuo H. 2016	low expression
JPX/Ma W.2016	low expression	MVIH/Yuan SX. 2012	high expression
kcnq1ot1/Shen J. 2015	high expression	PANDAR/Peng W. 2015	high expression
LINC00346/Zhang J.2015	high expression	PVT1/Ding C. 2014	high expression
LincRNA-p21/Ning Y.2015	low expression	PVT1/Wang F,YJ. 2015	high expression
LOC283663/Zhang J.2015	low expression	SNHG1/Zhang M.2016	high expression
LOC338651/Zhang J.2015	low expression	SNHG3/Zhang T. 2016	high expression
MAPKAPK5AS1/Zhang J.2015	high expression	UCA1/Kamel MM. 2016	high expression
MEG3/Zhuo H. 2016	low expression	WRAP53/Kamel MM.2016	high expression
MVIH/Yuan SX. 2012	high expression	ZEB-1-AS1/Li T. 2016	high expression
PANDAR/Peng W. 2015	high expression	AFAP1-AS1/Lu X. 2016	high expression
PCAT-1/Yan TH. 2015	high expression	CPS1-IT1/Wang TH. 2016	low expression
plncRNA-1/Dong L. 2016	high expression	Ftx/Liu Z. 2016	high expression
PRINS/Shen J. 2015	high expression	LincRNA-p21/Yang N.2015	low expression
PVT1/Ding C.2014	high expression	SNHG3/Zhang T. 2016	high expression
TUSC7/Wang Y. 2016	low expression		
