# Supplementary material for: lncRNAs as prognostic molecular biomarkers in hepatocellular carcinoma: a systematic review and meta-analysis
Source: Oncotarget. 2017 Jul 25;8(35):59638–47. doi: 10.18632/oncotarget.19559 (PMC5601763; doi:10.18632/oncotarget.19559)
Supplement: Supplementary file 2 [file oncotarget-08-59638-s002.doc]

| **Supplementary Table 1**: Summary table of main characteristics for the eligible studies | | | | | | | | | | | | | | |  |
| --- | --- | --- | --- | --- | --- | --- | --- | --- | --- | --- | --- | --- | --- | --- | --- |
| **Study(year)** | **origin of population** | | **NO. of patients(M/F)** | | **Age(y)** | | **NO. with lncRNA(H/L)** | | **lncRNA cutoff** | | **Survival analysis** | **follow-up** | **Hazard Ratios lncRNAs** | | **lncRNAs** |
| Yuan SX et al 2012 | China | 215(179/36) | | ≤55 | | 215（108/107） | | mean expression level | | OS/RFS | | 4-30 | SC | 32MVIH | |
| Peng W et al 2015 | China | 482(378/104) | | ≤60 | | 482(326/156) | | NR | | OS | | NR | SC | 14CCHE1,PANDAR | |
| Zhang JH et al 2016 | China | 152(92/60) | | ≥50 | | 152(77/75) | | mean expression level | | OS | | NR | SC | 34SNHG15 | |
| Liu ZK et al 2016 | China | 126(99/27) | | ＜65 | | 126(65/61) | | mean expression level | | OS/DFS | | NR | SC | 10Ftx | |
| Zhang T et al 2016 | China | 144(119/25) | | ≤55 | | 144(91/53) | | NR | | OS/RFS/DFS | | 60 | SC | 38SNHG3 | |
| Guo WX et al 2015 | China | 617(NR/NR) | | NR | | 617(NR/NR) | | NR | | OS/DFS | | NR | SC | 5ICR | |
| Li SP et al 2016 | China | 38(29/9) | | ＜60 | | 38(23/15) | | NR | | OS | | NR | SC | 8HULC | |
| Zhang M et al 2016 | China | 82(69/13) | | ≤55 | | 82(41/41) | | NR | | OS/RFS | | NR | SC | 37SNHG1 | |
| Kamel MM et al 2015 | Egypt | 160(108/52) | | ≥57 | | 160(NR/NR) | | 1.04 | | RFS | | 21.5 | SC | 7UCA1,7WRAP53 | |
| Dong LY et al 2016 | China | 84(65/19) | | ≤65 | | 84（48/36） | | mean expression level | | OS | | NR | SC | 3plncRNA-1 | |
| Xiao CH et al 2016 | China | 51(26/25) | | ＜60 | | 51（26/25） | | mean expression level | | RFS | | NR | SC | 27LINCRP1130-1 | |
| Qi HL et al 2015 | China | 40(31/9) | | NR | | 40(14/26) | | NR | | OS | | NR | SC | 16EGFR-AS1 | |
| Wang FQ et al 2015 | China | 97(58/39) | | ≤60 | | 97（60/37） | | NR | | OS/DFS | | NR | DE | 22CARLo-5 | |
| Lu X et al 2016 | China | 156(147/9) | | ＞51 | | 156（78/78） | | NR | | OS/DFS | | NR | SC | 11AFAP-AS1 | |
| Wang Y et al 2016 | China | 75(51/24) | | ≥50 | | 75（37/38） | | 0.33 | | OS/DFS | | NR | SC | 26TUSC7 | |
| Zhou T et al 2016 | China | 109(80/29) | | ≥60 | | 109（54/55） | | NR | | OS | | NR | SC | 39BANCR | |
| Lv L et al 2015 | China | 90(75/15) | | ＜60 | | 90(45/45) | | NR | | OS | | NR | SC | 12WT1-AS | |
| Yang Z et al 2011 | China | 60(55/5) | | ≤50 | | 60(32/28) | | NR | | RFS | | 18.6 | SC | 30HOTAIR | |
| Shen J et al 2015 | Caucasian/African-American/Hispanic/Asian/Other | 65（49/16） | | ≥60 | | 65(NR/NR) | | 0.5 probability of death | | OS | | NR | DE | 1821A,18BACE1AS,18kcnq1ot1,18PRINS,18SNHG4,18Tmevpg1,18UCA1 | |
| Chang L et al 2015 | China | 50(37/13) | | ≥65 | | 50（25/25） | | NR | | OS | | NR | SC | 1GAS5 | |
| Ma WJ et al 2016 | China | 100(77/23) | | ≥50 | | 100(NR/NR) | | NR | | OS | | NR | SC | 13JPX,13XIST | |
| Quagliata L et al 2014 | Switzerland | 52(47/5) | | 70.5 | | 52(32/20) | | NR | | OS | | NR | SC | 4HOTTIP | |
| Tu ZQ et al 2014 | China | 71(40/31) | | ＜60 | | 71(20/51) | | mean expression level | | OS | | 60 | SC | 21GAS5 | |
| Hua L et al 2015 | China | 92(53/39) | | ＜60 | | 92(46/46) | | NR | | OS | | NR | SC | 6ANRIL | |
| Yan TH et al 2015 | China | 117(68/49) | | ＞50 | | 117（59/58） | | NR | | OS | | NR | SC | 28PCAT-1 | |
| Shi XM et al 2015 | China | 84(53/31) | | ≥50 | | 84(42/42) | | NR | | OS | | NR | SC | 19Sox2ot | |
| Zhang JY et al 2016 | China | 77(58/19) | | ＜60 | | 77(56/21) | | NR | | OS | | NR | SC | 35AFAP1-AS1 | |
| Peng W et al 2016 | China | 112(55/57) | | ＜60 | | 112(54/58) | | NR | | OS | | NR | SC | 15PANDAR | |
| Wang TH et al 2016 | China | 119(101/18) | | ＞55 | | 119（57/62） | | NR | | OS/DFS | | NR | SC | 25CPS1-IT1 | |
| Yuan SX et al 2016 | China | 135（NR/NR） | | NR | | 135(NR/NR) | | NR | | OS/RFS | | NR | DE | 31DANCR | |
| Sui CJ et al 2016 | China | 70(59/11) | | ≤50 | | 70(35/35) | | mean expression level | | OS/RFS | | NR | SC | 20GIHCG | |
| Zhuo H et al 2016 | China | 72(59/13) | | ＜60 | | 72(36/36) | | mean expression level | | OS/RFS | | NR | SC | 40MEG3 | |
| Ding CF et al 2015 | China | 58(53/5) | | ≤60 | | 58(49/9) | | NR | | OS/RFS | | 27.58 | DE | 2PVT1 | |
| Li T et al 2016 | China | 102(73/29) | | ≤50 | | 102(51/51) | | mean expression level | | OS/RFS | | NR | SC | 9ZEB-1-AS1 | |
| Wang F et al 2015 | China | 98(85/13) | | ＜55 | | 98（49/49） | | NR | | OS | | NR | DE | 23CARLo-5,23PVT1,23UCA1 | |
| Yang N et al 2015 | China | 70(49/21) | | NR | | 70(42/38) | | NR | | OS/DFS | | NR | SC | 29LincRNA-p21 | |
| Ge YX et al 2015 | China | 48(42/6) | | 57 | | 48(NR/NR) | | NR | | OS | | 18 | SC | 17HOTTIP | |
| Zhang J et al 2015 | China | 322(NR/NR) | | NR | | 322(NR/NR) | | NR | | OS | | NR | SC | 36CECR7,36FLJ90757,36LINC00346,36LOC283663,36LOC338651,36MAPKAPK5AS1 | |
| Zhang DY et al 2016 | China | 49(45/4) | | ≤55 | | 49(33/16) | | 3.98 | | OS | | NR | DE | 33SNHG20 | |
| Wang F et al 2014 | China | 89(73/16) | | ≤55 | | 89(44/45) | | mean expression level | | OS/RFS | | NR | SC | 24PVT1 | |
| **H:**high,L:low,OS:overall survival,RFS: recurrence-free survival,DFS:disease-free survival, DE:data extrapolated,SC:survival curve;NR:not reported. | | | | | | | | | | | | | | |  |
|  |
