# Supplementary material for: lncRNAs as prognostic molecular biomarkers in hepatocellular carcinoma: a systematic review and meta-analysis
Source: Oncotarget. 2017 Jul 25;8(35):59638–47. doi: 10.18632/oncotarget.19559 (PMC5601763; doi:10.18632/oncotarget.19559)
Supplement: Supplementary file 3 [file oncotarget-08-59638-s003.doc]

| **Supplementary Table 2:** Summary table of HRs and their 95% CI. | | | | |
| --- | --- | --- | --- | --- |
| **Study(year)** | **HR** | **95%CI(LL-UL)** | **p-value^a^** | **outcome^b^** |
| 21A/Shen J.2015 | 0.4 | 0.15-1.06 | 0.056 | OS |
| AFAP-AS1/Lu X. 2016 | 1.82 | 1.03-3.22 | 0.004 | OS |
| AFAP-AS1/Zhang J Y. 2016 | 1.47 | 0.90-2.40 | 0.029 | OS |
| ANRIL/Hua L. 2015 | 2.68 | 1.26-5.73 | 0.003 | OS |
| BACE1AS/Shen J. 2015 | 0.5 | 0.20-1.23 | 0.114 | OS |
| BANCR/Zhou T. 2016 | 2.08 | 1.35-3.21 | <0.001 | OS |
| CARLo-5/Wang F,XC. 2015 | 2.98 | 1.72-5.17 | 0.011 | OS |
| CCHE1/Peng W. 2016 | 0.98 | 0.29-3.24 | 0.041 | OS |
| CECR7/Zhang J. 2015 | 2.11 | 1.39-3.20 | 0.009 | OS |
| CPS1-IT1/Wang TH. 2016 | 0.57 | 0.34-0.97 | 0.042 | OS |
| DANCR/Yuan SX. 2016 | 0.36 | 0.18-0.72 | 0.004 | OS |
| EGFR-AS1/Qi HL. 2016 | 2.7 | 0.49-14.94 | <0.05 | OS |
| FLJ90757/Zhang J. 2015 | 0.62 | 0.46-0.84 | 0.025 | OS |
| Ftx/Liu Z. 2016 | 1.84 | 1.17-2.90 | 0.0003 | OS |
| GAS5/Chang L. 2016 | 0.31 | 0.16-0.60 | <0.001 | OS |
| GAS5/Tu ZQ. 2014 | 0.42 | 0.26-0.66 | 0.002 | OS |
| GIHCG/Sui CJ. 2016 | 1.86 | 0.77-4.50 | 0.0276 | OS |
| HOTTIP/Ge Y. 2015 | 2.35 | 1.45-3.82 | 0.018 | OS |
| HOTTIP/Quagliata L.2014 | 2.41 | 1.32-4.41 | <0.05 | OS |
| HULC/Li SP. 2016 | 3.84 | 1.72-8.75 | <0.001 | OS |
| ICR/Guo WX. 2016 | 3.03 | 2.25-4.08 | <0.001 | OS |
| JPX/Ma W.2016 | 0.44 | 0.22-0.88 | 0.015 | OS |
| kcnq1ot1/Shen J. 2015 | 3 | 1.01-8.95 | 0.044 | OS |
| LINC00346/Zhang J.2015 | 1.87 | 1.42-2.47 | 0.002 | OS |
| LincRNA-p21/Yang N.2015 | 0.47 | 0.27-0.82 | 0.0024 | OS |
| LOC283663/Zhang J.2015 | 0.88 | 0.64-1.20 | 0.021 | OS |
| LOC338651/Zhang J.2015 | 0.62 | 0.44-0.87 | 0.036 | OS |
| MAPKAPK5AS1/Zhang J.2015 | 1.48 | 1.12-1.96 | 0.032 | OS |
| MEG3/Zhuo H. 2016 | 0.64 | 0.27-1.53 | 0.012 | OS |
| MVIH/Yuan SX. 2012 | 1.85 | 1.05-3.27 | 0.033 | OS |
| PANDAR/Peng W. 2015 | 1.83 | 1.50-2.24 | <0.0001 | OS |
| PCAT-1/Yan TH. 2015 | 2.98 | 1.11-7.96 | 0.001 | OS |
| plncRNA-1/Dong L. 2016 | 1.87 | 1.05-3.35 | 0.02 | OS |
| PRINS/Shen J. 2015 | 1.7 | 0.62-4.66 | 0.308 | OS |
| PVT1/Ding C.2014 | 1.05 | 0.69-1.60 | 0.464 | OS |
| PVT1/Wang F,YJ.2015 | 1.93 | 0.90-4.13 | 0.0104 | OS |
| SNHG1/Zhang M.2016 | 2.25 | 1.04-4.87 | 0.0068 | OS |
| SNHG15/Zhang JH.2016 | 2.25 | 1.04-4.87 | 0.001 | OS |
| SNHG20/Zhang D. 2016 | 3.98 | 1.98-8.02 | 0.0001 | OS |
| SNHG3/Zhang T. 2016 | 3.46 | 1.82-6.59 | 0.0001 | OS |
| SNHG4/Shen J. 2015 | 0.38 | 0.14-1.06 | 0.064 | OS |
| Sox2ot/Shi XM | 2.64 | 1.33-5.27 | 0.001 | OS |
| Tmevpg1/Shen J.2015 | 0.59 | 0.24-1.45 | 0.267 | OS |
| TUSC7/Wang Y. 2016 | 0.29 | 0.12-0.71 | 0.007 | OS |
| UCA1/Shen J. 2015 | 0.5 | 0.20-1.24 | 0.134 | OS |
| UCA1/Wang F,YH.2015 | 1.86 | 1.08-3.21 | 0.026 | OS |
| WT1-AS/Lv L. 2015 | 0.65 | 0.32-1.32 | 0.018 | OS |
| XIST/Ma W. 2016 | 0.45 | 0.24-0.86 | 0.017 | OS |
| ZEB-1-AS1/Li T. 2016 | 1.77 | 1.00-3.13 | 0.017 | OS |
| CARLo-5/Wang F,XC. 2015 | 1.81 | 0.99-3.30 | 0.01 | RFS |
| DANCR/Yuan SX. 2016 | 0.45 | 0.27-0.74 | 0.001 | RFS |
| GIHCG/Sui CJ. 2016 | 1.6 | 0.75-3.42 | - | RFS |
| HOTAIR/Yang Z.2011 | 3.56 | 1.67-7.63 | 0.04 | RFS |
| HULC/Li SP. 2016 | 3.42 | 1.64-7.12 | 0.001 | RFS |
| LINCRP1130-1/Xiao C. 2016 | 0.72 | 0.20-2.56 | 0.017 | RFS |
| MEG3/Zhuo H. 2016 | 0.55 | 0.25-1.20 | - | RFS |
| MVIH/Yuan SX. 2012 | 1.99 | 1.31-3.00 | 0.001 | RFS |
| PANDAR/Peng W. 2015 | 1.34 | 1.09-1.64 | - | RFS |
| PVT1/Ding C. 2014 | 1.27 | 0.80-2.20 | - | RFS |
| PVT1/Wang F,YJ. 2015 | 1.81 | 0.95-3.46 | - | RFS |
| SNHG1/Zhang M.2016 | 2.08 | 1.16-3.73 | - | RFS |
| SNHG3/Zhang T. 2016 | 2.25 | 1.16-4.36 | - | RFS |
| UCA1/Kamel MM. 2016 | 2.2 | 0.86-5.63 | 0.099 | RFS |
| WRAP53/Kamel MM.2016 | 4.13 | 1.75-9.74 | 0.001 | RFS |
| ZEB-1-AS1/Li T. 2016 | 2.17 | 1.34-3.52 | - | RFS |
| AFAP1-AS1/Lu X. 2016 | 2.1 | 1.25-3.52 | - | DFS |
| CPS1-IT1/Wang TH. 2016 | 0.55 | 0.34-0.88 | 0.011 | DFS |
| Ftx/Liu Z. 2016 | 2.18 | 1.43-3.33 | - | DFS |
| LincRNA-p21/Yang N.2015 | 0.5 | 0.29-0.85 | - | DFS |
| SNHG3/Zhang T. 2016 | 2.72 | 1.26-5.88 | - | DFS |
| TUSC7/Wang Y. 2016 | 0.34 | 0.14-0.82 | 0.015 | DFS |
| **^a^:** Reported in the article,**^b^:**The important prognostic factors. | | | | |
